# Consecutive monoculture of sweet potato reduces yield due to deteriorated soil health and disrupted nutrient cycling

**DOI:** 10.3389/fmicb.2026.1747390

**Published:** 2026-04-30

**Authors:** Can Chen, Yini Shi, Manman Zheng, Sitong Han, Ju Zhang, Zhongke Sun, Zonghao Yue

**Affiliations:** 1 Key Laboratory of Plant Genetics and Molecular Breeding, Henan Key Laboratory of Crop Molecular Breeding and Bioreactor, Zhoukou Normal University, Zhoukou, China; 2 College of Life Sciences and Agronomy, Zhoukou Normal University, Zhoukou, China; 3 School of Biological Engineering, Henan University of Technology, Zhengzhou, China

**Keywords:** consecutive monoculture, high-throughput amplicon sequencing, microbial diversity, soil microbiome, sweet potato

## Abstract

Sweet potato (*Ipomoea batatas* L.) is a staple food with many promising health benefits. However, obstacles associated with continuous cropping are common in modern intensive sweet potato production, partially due to imbalances in the soil microbiome. This research investigates the succession of the soil microbiome and its impacts following 1, 3, and 5 years of consecutive sweet potato monoculture. The results showed significantly higher diversity and homogeneity in bacteria than in fungi. Although a large proportion of operational taxonomic units (OTUs) were shared, richness analysis indicated a significant decrease in the total number of OTUs for both bacteria and fungi, especially in the fifth year. At the phylum level, Firmicutes and Parcubacteria significantly decreased, while Cryptomycota significantly increased (*p* < 0.05). Further analysis of the prokaryotic community using BugBase and the Functional Annotation of Prokaryotic Taxa (FAPROTAX) database showed significant changes in many phenotypes and functions. In particular, alterations in human pathogens and *Cyanobacteria*, as well as differences in carbon metabolism and nitrogen conversion in soils, were revealed for the first time. It was also observed that the contents of soil organic matter (OM), total carbon (TC), and total nitrogen (TN) had a linear correlation with the abundance of *Cyanobacteria*. However, yield was positively correlated with soil pH but negatively correlated with disease incidence. Taken together, in addition to the distinct succession of the microbial community structure, the study indicates that consecutive monoculture of sweet potato has a significant impact on the health status of soil and nutrient cycling.

## 1 Introduction

As a rich source of vitamins A and C, the sweet potato (*Ipomoea batatas* L.) tuber is one of the world’s most important food crops, cultivated across tropical and subtropical regions, particularly in Asia, Africa, and the Pacific (Sheikha and Ray, 2017; Alam, 2021; Escobar-Puentes et al., 2022; Jiang et al., 2024). China, the largest producer in the world, yielded approximately 51.4 million tons of sweet potatoes in 2023 (FAOSTAT, 2023). In addition to the consumption of fresh tender leaves, stems, and roots, the large, starchy, sweet-tasting tuberous roots of sweet potato are widely processed for the production of starch noodles in Asia (Xiang et al., 2018; Kim et al., 2020). Meanwhile, it is widely recognized that sweet potato is not simply a staple food but also a valuable medicinal plant, possessing diverse chemical constituents and exhibiting various health benefits and biological activities (Wang et al., 2016; Luo et al., 2021; Laveriano-Santos et al., 2022; Ivane et al., 2024). Given its significant role in human nutrition and health, it is essential to enhance sweet potato yield and manage its pests and diseases. Soil plays a pivotal role in sweet potato growth due to its influential impacts on the development of storage roots, mainly through the supply of nutrients and interactions with microorganisms (Mukhongo et al., 2017; Yao et al., 2020; Abd El-Mageed et al., 2022). The vast number of microorganisms in soil, collectively termed the soil microbiome, constitutes a dynamic microecosystem that drives and fine-tunes several biological processes (Bhattacharjee et al., 2022). Steering the soil microbiome can not only increase yield but also inhibit soil-borne diseases and reduce pollution by improving soil immunity and resilience (Wang and Li, 2019; Yang et al., 2023; García-Serquén et al., 2024). To explore the soil microbiome for sustainable agriculture, it is necessary to characterize the soil microbiome community and identify the factors that shape soil microbial structures across space and time (Fierer, 2017). Although predictability exhibits taxonomic and spatial-scale dependence, advances in metagenomic analyses have greatly expanded our understanding of the soil microbiome (Averill et al., 2021). For sweet potato, only a few studies have investigated the soil microbiome using either culture-dependent or culture-independent techniques. A pioneering study using denaturing gradient gel electrophoresis (DGGE) and pyrosequencing analysis of the 16S rRNA gene elucidated the impact of sweet potato genotypes on the composition of rhizosphere prokaryotes, a special part of the soil microbiome (Marques et al., 2014). This study also noted differences in bacterial community composition between the surrounding bulk soil and the rhizospheric soil of sweet potato. This finding was also confirmed by a more comprehensive study that quantified relative changes in microbiome composition across the storage root skin, rhizosphere, and surrounding soil of sweet potato (Pepe-Ranney et al., 2020). To control pathogens that lead to disease and yield reduction, many biocontrol agents have been introduced into soils. For example, the fungu*s Plenodomus destruens*, which causes foot rot disease, can be effectively inhibited by *Bacillus safensis* and *Bacillus velezensis*. As shown by DGGE analysis, the soil microbial community (both bacteria and fungi) associated with sweet potato in response to *Bacillus* sp. application seems to be strain-specific (Mateus et al., 2019).

In addition, continuous cropping has become the most common system in modern intensive agricultural production; however, obstacles often arise after a few years of consecutive monoculture (Tan et al., 2021). In practice, it is recognized that continuous cropping obstacles are common in sweet potato and severely limit yield and quality (Gu et al., 2022). Therefore, the soil microbiome might be a key factor in addressing continuous cropping obstacles in sweet potato. For example, the decline in soil quality after successive sweet potato monoculture can be partially attributed to imbalances in soil microbial communities, as shown in a pyrosequencing study of soil bacterial communities (Li et al., 2019). Furthermore, two studies separately revealed changes in fungal and bacterial community structures in rhizospheric soil after continuous cropping of sweet potato for 4 years (Gao et al., 2019; Gao et al., 2021). However, the rhizosphere compartments of sweet potato differ significantly from each other and the surrounding soil; for example, the rhizosphere and surrounding soils harbor distinct microbial communities (Pepe-Ranney et al., 2020). Moreover, as widely acknowledged, the soil microbiome associated with a certain plant often varies with plant genotype, growth stage, and soil type. To address the problem of continuous cropping obstacles in sweet potato, the current study investigates changes in the soil microbial community, soil health status, and nutrient cycling at the harvest stage using high-throughput amplicon sequencing of a popular sweet potato cultivar after 5 years of consecutive monoculture.

## 2 Materials and methods

### 2.1 Soil sample collection

Soil samples were randomly collected from a major sweet potato planting region (approximately 33°68′89″N, 115°12′37″E) by Henan Tianyu Sweet Potato and Food Products Co., Ltd., located in Dancheng County, Zhoukou City, China. This region has an average annual temperature of 14.6 °C and a mean annual precipitation of 738.6 mm. Shangshu 19 is a high-yield cultivar that is relatively resistant to continuous cropping. On 20th October 2017 (just before harvest), soil samples were carefully collected into sterile tubers from five different sites (10 cm below the surface, and ~2 g wet weight) in fields planted with Shangshu 19 for 1, 3, and 5 years. A total of three agricultural fields (biological replicates) planted with the indicated sweet potato cultivar were sampled. For each field, soil samples were randomly collected from five sites. These five samples collected from the same field were mixed to obtain a composite soil sample, which was considered an independent sample. After being immediately transported to the laboratory, 1 g of soil from each site within the same year was mixed to form a composite sample. Samples from fields under consecutive cropping for different years were labeled S1, S3, and S5, respectively. In total, three repeats for each year were used (S1_1, S1_2, and S1_3 classified as G1; S3_1, S3_2, and S3_3 classified as G3; and S5_1, S5_2, and S5_3 classified as G5).

### 2.2 Sample processing and amplicon sequencing

The samples were passed through a 2 mm sieve and stored at −78 °C for microbial analysis. Microbial DNA was extracted from the samples using the E. Z. N. A.^®^ Soil DNA Kit (Omega Bio-tek, GA, United States) according to the manufacturer’s protocols. The 16S rRNA gene was amplified using polymerase chain reaction (PCR) with the primers 338F/806R (Xu et al., 2016). The 18S rRNA gene was amplified using the primers SSU0817F/1196R (Rousk et al., 2010). PCR amplification, as well as the extraction and purification of amplicons, were performed according to standard protocols described elsewhere, using TransStart FastPfu DNA Polymerase (TransGen Biotech Co., Beijing, China) on an ABI GeneAmp® 9700 thermal cycler (Yang et al., 2018). Purified PCR products were quantified using the QuantiFluor® ONE dsDNA System (Promega). Single-stranded DNA libraries were prepared using the TruSeqTM DNA Sample Prep Kit (Illumina Inc., CA, United States). The pooled DNA product was used to construct an Illumina paired-end library following Illumina’s genomic DNA library preparation protocol. Then, the amplicon library was sequenced using paired-end (2 × 300) sequencing on an Illumina MiSeq PE300 platform by a commercial service provider (Shanghai Majorbio Co., Ltd.) following standard procedures.

### 2.3 Sequencing data analysis and deposition

High-quality sequences were obtained through quality control and filtering using Fastp 0.19.6.^1^ Operational taxonomic units (OTUs) were clustered at a 97% similarity cutoff using UPARSE (version 7.1^2^) with the SILVA database (Release 138^3^). Chimeric sequences were removed using UCHIME. The data were analyzed using the Majorbio Cloud platform^4^ with default settings, according to the provided instructions (Ren et al., 2022). The complete raw sequencing data for 16S rRNA and 18S rRNA were deposited in the NCBI Sequence Read Archive database and are publicly available under BioProject accession number PRJNA895716.

### 2.4 Bioinformatic prediction of bacterial phenotypes and functions

Considering the richness of bacteria and their significant impacts on soil fertility and plant health, microbial phenotypes were predicted using BugBase.^5^ To further explore how changes in the bacterial community influence soil biochemistry, the Functional Annotation of Prokaryotic Taxa (FAPROTAX) database was used to predict alterations in potential functions during continuous cropping (Louca et al., 2016).

### 2.5 Assay of soil physiochemical properties

The pH was measured using a pH meter in the slurry at a constant soil-to-water ratio of 1:2.5. The K_2_Cr_2_O_7_–H_2_SO_4_ oxidation approach was adopted to assess the organic matter (OM) content in the soil. Total carbon (TC) and total nitrogen (TN) in the soils were determined through digestion, distillation, and titration procedures using an elemental analyzer (Thermo Scientific™, United States). Available phosphorus (AP) was extracted using ammonium fluoride and hydrochloric acid (Liu et al., 2016) and measured using the molybdenum blue method with a UV photometer (Spectrum lab 24, Shanghai, China). Available potassium (K+) was determined by flame photometry after NH_4_OAc neutral extraction (Li et al., 2022). Other soil properties were determined as described elsewhere (Gao and Xie, 2023).

### 2.6 Measurement of yield and disease incidence

After harvest, the disease incidence rate was calculated as described by Li et al. (2019). Briefly, three fields located in Dancheng County, planted with sweet potato for 0, 2, and 4 years, respectively, were used as experimental sites. Sweet potato (cultivar Shangshu 19) was planted on 10th May and harvested on 20th October 2019. Agronomic management and fertilization regimes were the same for all three fields. This included a planting density of 50,000 plants/ha and fertilizer amendment (N 90 kg/hm^2^, P_2_O_5_ 75 kg/hm^2^, and K_2_O 90 kg/hm^2^). Each field had an area of 300 m^2^ (10 m × 30 m), and three representative subplots within each field with an area of 2 m × 2 m were randomly selected for the estimation of disease incidence and crop yield. In each subplot (4 m^2^), all sweet potato tubers were carefully collected after harvest. Any tubers with visible black spots or partial rot were recorded as infected tubers. The incidence rate was defined as the percentage of infected tubers relative to the total number of tubers in each subplot. All tubers collected from each subplot were rinsed with tap water and weighed to estimate yield.

### 2.7 Data processing and correlation analysis

Disease incidence was calculated as the mean of data from the three subplots. For yield, clean fresh tubers from the three subplots were weighted and expressed as t/hm^2^. All data are presented as the mean of at least three replicates. Significance was determined using the SPSS Statistics 16.0 software with one-way ANOVA followed by Duncan’s test, and significant differences were defined at a *p*-value of <0.05 or a *p*-value of <0.01. Principal component analysis (PCA) and analysis of similarities (ANOSIM) based on the Bray–Curtis dissimilarity index were used to differentiate the samples. Linear discriminant analysis effect size (LEfSe) was used to identify significant variations among different samples (LDA > 3.5, *p* < 0.05). Heatmaps of Pearson correlation coefficients were generated for the evaluation of the relationship between the microbial community and physicochemical properties (0.7 ≤ |Pearson’s *r*| ≤ 1) using the Majorbio Cloud platform (see Footnote 4).

## 3 Results

### 3.1 Monoculture of sweet potato changes soil microbiome structure

**Figure 1:**
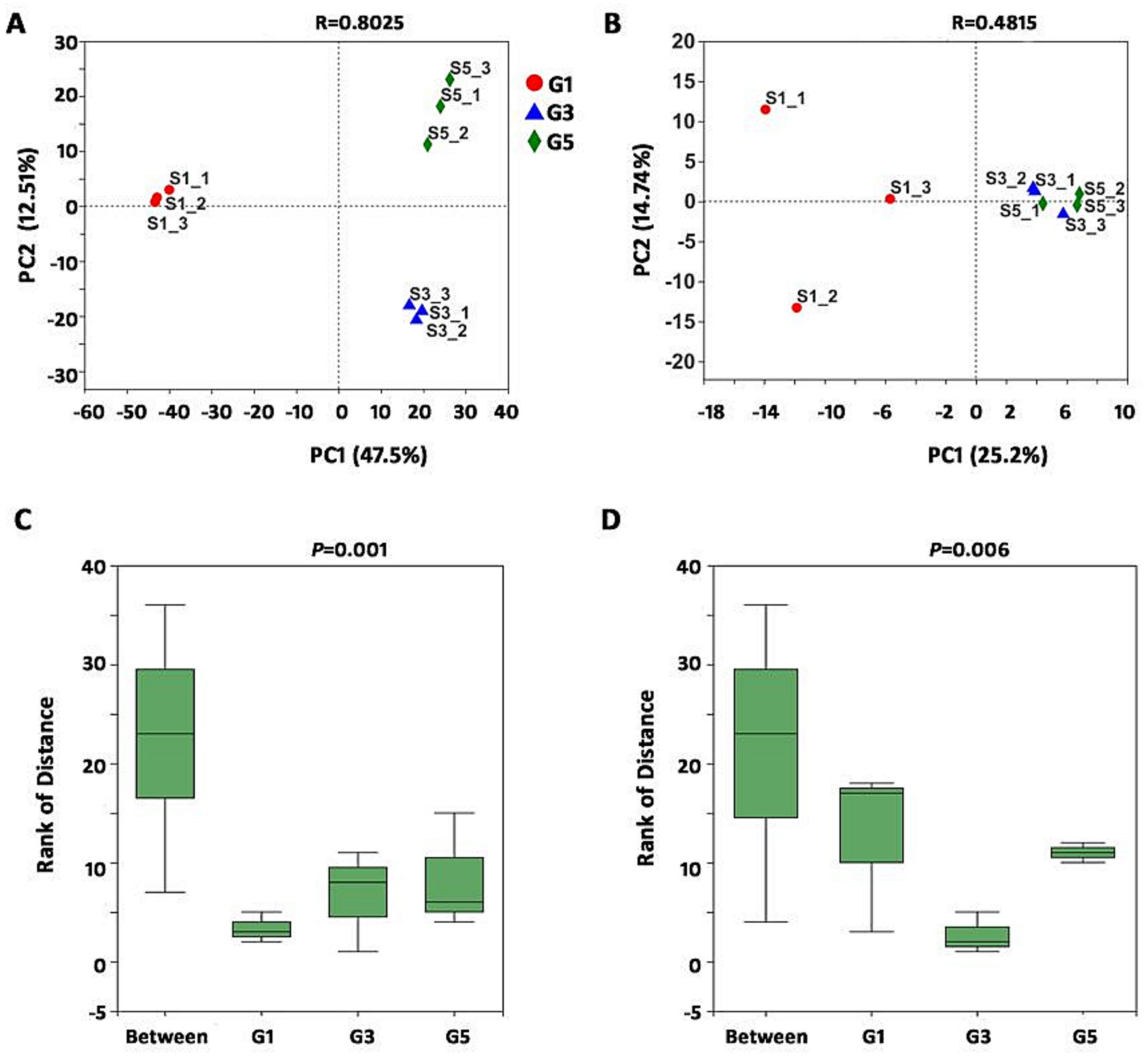
Comparison of nine samples using PCA and ANOSIM among three sample groups at the OTU level. **(A)** PCA of prokaryotic microbiota among the nine samples classified into three groups. **(B)** PCA of fungi among the nine samples classified into three groups. **(C)** Distance box plot of prokaryotic OTUs among the three sample groups. **(D)** Distance box plot of fungal OTUs among the three sample groups. ANOSIM was conducted using the Bray–Curtis method with 999 permutations. PCA, principal component analysis; ANOSIM, analysis of similarities; OTU, operational taxonomic unit.

The coverage of all samples was above 99%, indicating that the sequencing depth met the requirements of the experiment. After quality control, a total of 323,292 16S rRNA gene sequences with a mean length of 441 bp and 328,460 18S rRNA gene sequences with a mean length of 401 bp were obtained from nine samples, respectively (Supplementary Table S1). With a 3% dissimilarity threshold, the obtained sequences were then clustered into 2,114 operational taxonomic units (OTUs), representing 34 phyla and 249 genera for prokaryotes. For fungi, 268 OTUs were identified, representing 37 phyla and 89 genera, after assigning their taxonomy using the Ribosomal Database Project Classifier. At the OTU level, principal component analysis (PCA) suggested that the G1, G3 and G5 groups were distinctly clustered in terms of prokaryotic microbial community composition (Figure 1A). Although clustering between G3 and G5 was not distinct, the three groups were separately clustered in terms of fungal community composition (Figure 1B). Further ANOSIM using the Bray–Curtis dissimilarity index indicated significant differences in the structure of both prokaryotes (*R* = 0.918, *p* = 0.001) and fungi (*R* = 0.72, *p* = 0.006) among the three groups (Figures 1C,D). Therefore, samples collected from the study areas showed clear differences in terms of the soil microbiome after consecutive monoculture over different years.

### 3.2 Monoculture of sweet potato decreases soil microbial diversity and richness

**Figure 2:**
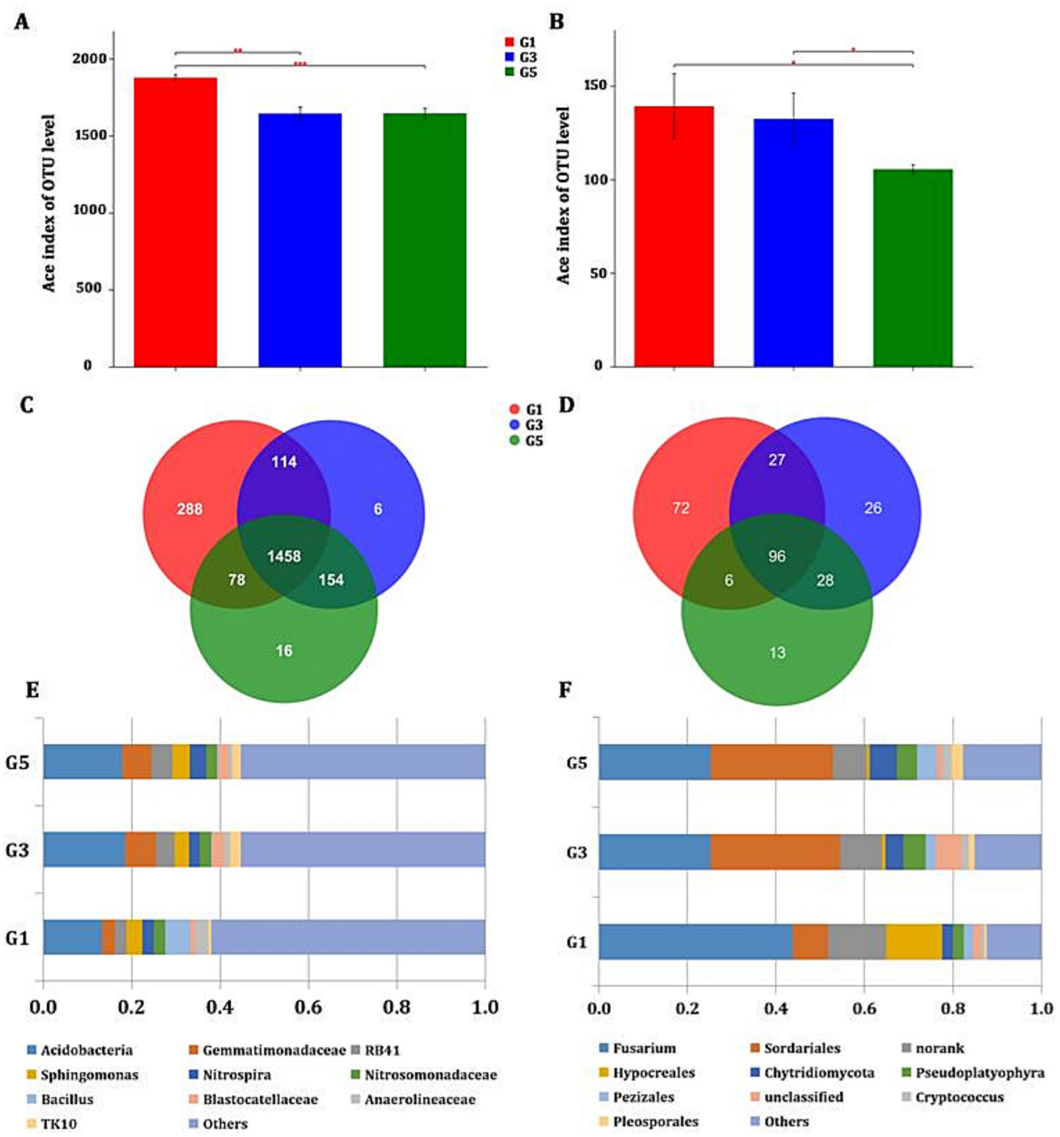
Analysis of diversity and community composition among three groups. **(A)** Alpha diversity of prokaryotic microbiota. **(B)** Alpha diversity of fungi. **(C)** Venn diagram of prokaryotic OTUs. **(D)** Venn diagram of fungal OTUs. **(E)** Community bar plot of prokaryotic microbiota. **(F)** Community bar plot of fungi. Comparison of alpha diversity among multiple groups was performed using Student’s *t*-test based on the ACE index at the OTU level. Community abundance is presented as a relative percentage at the genus level. Only the top 10 genera are shown in **(E,F)**. OTU, operational taxonomic unit; *, 0.01 < *p* ≤ 0.05; **, 0.001 < *p* ≤ 0.01; ***, *p* ≤ 0.001.

For prokaryotic microbiota, 1,938, 1,732, and 1,706 OTUs were found in the G1, G3, and G5 groups, respectively (Figure 2A). According to the ACE estimator, groups G3 and G5 were significantly different from G1 (G1/G3, *p* = 0.001033; G1/G5, *p* = 0.000463). In contrast, only 201, 177, and 143 OTUs were found for eukaryotic fungi, and groups G1 and G5 were significantly different from G3 (G1/G5, *p* = 0.02908; G3/G5, *p* = 0.02953) (Figure 2B). Among the three groups, 1,458 OTUs representing prokaryotic microbiota and 96 OTUs representing fungi were shared (Figures 2C,D). At the phylum level, the dominant bacterial groups were Proteobacteria, Acidobacteria, Actinobacteria, Gemmatimonadetes, Chloroflexi, Bacteroidetes, Nitrospirae, Firmicutes, Planctomycetes, and Latescibacteria, while the most abundant fungal groups were Ascomycota, Ciliophora, Chytridiomycota, Basidiomycota, and Cryptomycota (Supplementary Figure S1). Compared to G1, Firmicutes and Parcubacteria significantly decreased, while Cryptomycota significantly increased in G3 and G5. To be more specific, the top 10 most abundant genera (ranked from high to low by proportion) were identified for bacteria (Figure 2E, e.g., Acidobacteria, *Gemmatimonadaceae*, and *Sphingomonas*) and fungi (Figure 2F, e.g., *Fusarium*, *Sordariales*, and *Hypocreales*).

As shown by the Kruskal–Wallis test at the phylum level in Supplementary Figure S2, many bacterial genera, including *Bacillus* and *Xanthomonadales*, significantly decreased, while *Gemmatimonadaceae*, *Gemmatimonas*, and *Haloangium* significantly increased after continuous cropping (0.001 < *p* ≤ 0.01). In contrast, *Cryptococcus*, *Chaetothyriales*, and *Orbiliaceae* significantly increased among fungi (*p* < 0.05). In addition, as many as 27 bacterial genera present in G1 were absent in G3 and G5 after consecutive monoculture (Supplementary Table S2). These results suggest that the soil microbial community was altered after continuous monoculture of sweet potato.

### 3.3 Monoculture of sweet potato alters bacterial functions related to pathogenesis and nutrient cycling

Among all nine phenotypes classified by BugBase, five (mobile element-containing, biofilm-forming, pathogenic, and stress-tolerant) showed no significant differences among the groups. Gram-negative bacteria significantly increased after 5 years of monoculture, while Gram-positive bacteria significantly decreased only in soils after 3 years of consecutive monoculture (Supplementary Figures S3A,B). In addition, the aerobic phenotype significantly increased, but the facultatively anaerobic phenotype significantly decreased (Supplementary Figures S3C,D).

**Figure 3:**
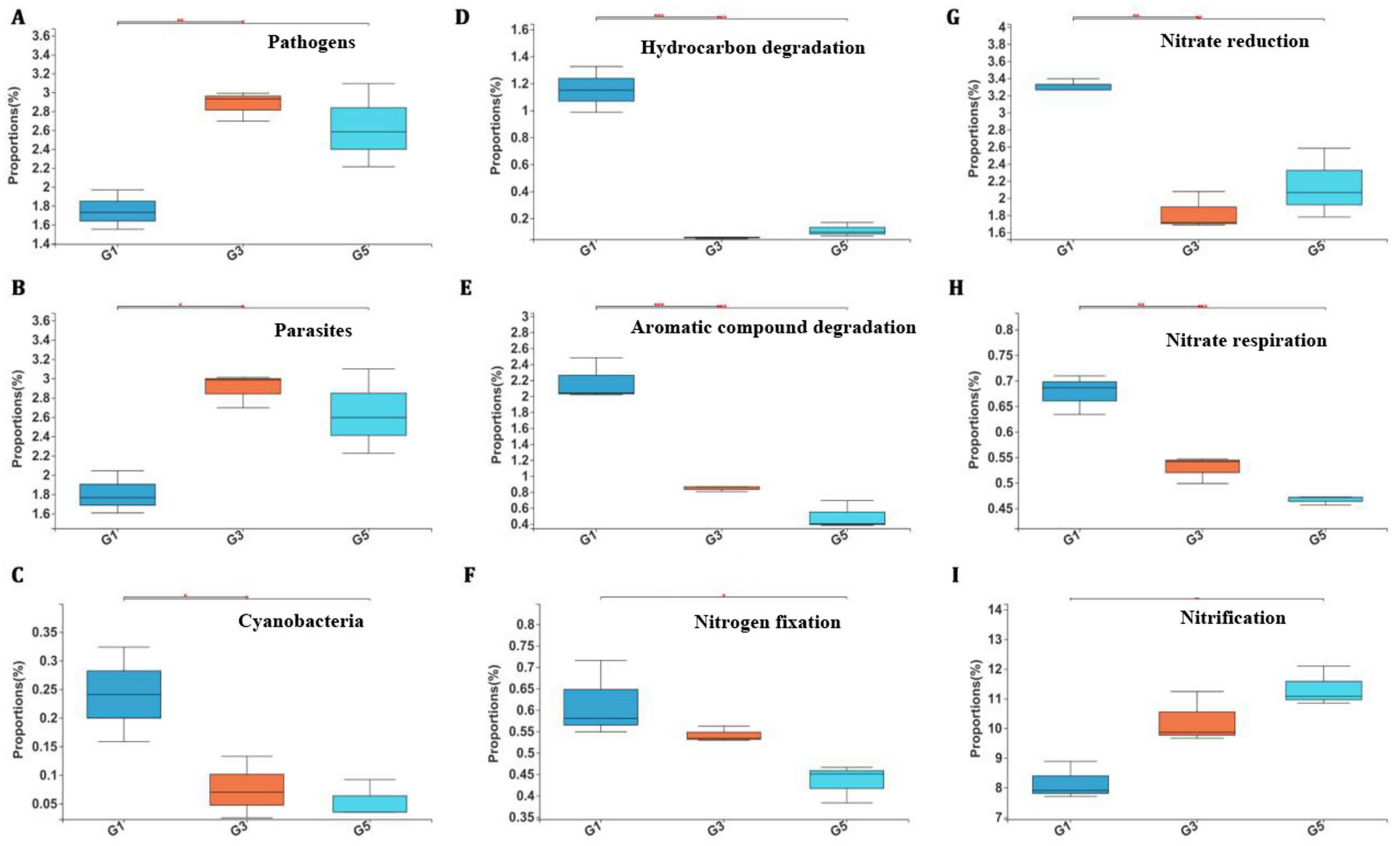
**(A)** Relative phenotype abundance of pathogens. **(B)** Relative phenotype abundance of parasites. **(C)** Relative phenotype abundance of Cyanobacteria. **(D)** Relative phenotype abundance of hydrocarbon degradation. **(E)** Relative phenotype abundance of aromatic compound degradation. **(F)** Relative phenotype abundance of Nitrogen fixation. **(G)** Relative phenotype abundance of nitrate reduction. **(H)** Relative phenotype abundance of nitrate respiration. **(I)** Relative phenotype abundance of nitrification. Analysis of bacterial phenotypes using BugBase among three groups at the genus level.

To further explore the potential impact of bacterial community changes, FAPROTAX was used to predict alterations in potential functions during continuous cropping. A total of 47 functional group assignments were obtained for 2,114 OTUs, of which 423 OTUs (20.01%) were assigned to at least one group, while the remaining 1,691 OTUs (79.99%) could not be assigned to any group (Supplementary Table S3). Chemoheterotrophy was the most abundant functional group (241 OTUs) in the bacterial community, within which aerobic chemoheterotrophy accounted for 210 OTUs. The Kruskal–Wallis H test showed an increase in functions related to human pathogens (Figure 3A) and animal parasites or symbionts (Figure 3B) in G3 and G5. These bacterial genera mainly included *Nocardia*, *Roseomonas*, *Limnobacter*, *Caenimonas*, *Methylibium*, *Acinetobacter*, and *Stenotrophomonas*. The majority of them overlapped between these two functional groups. The proportions of bacteria belonging to the *Cyanobacteria* group, including *Obscuribacterales* and *Subsection III*, were significantly decreased in G3 and G5 soils by approximately two- and four-fold, respectively (Figure 3C). The functions associated with hydrocarbon degradation, aromatic compound degradation, and nitrogen fixation also decreased in soils during consecutive monoculture of sweet potato (Figures 3D–F). There were five identified bacterial genera associated with nitrogen fixation, including *Hydrogenispora*, *Bradyrhizobium*, *Rhizobium*, *Azospirillum*, and *Skermanella*. A total of 54 OTUs, mainly composed of *Nitrospira* and *Nitrosomonadaceae*, associated with nitrification increased from 8.165% in G1 to 10.26% in G3 and 11.34% in G5 (Figure 3I). The increase in nitrification can be partially due to the significant decrease in both nitrate reduction and respiration (Figures 3G,H).

### 3.4 Monoculture results in yield loss and disease occurrence in sweet potato

Based on the calculation, sweet potato yield decreased with continuous monoculture, and the reduction was significant in the fifth year (Table 1). In contrast, disease incidence increased sharply even in the third year. After five years of continuous monoculture, the percentage of infected tubers reached nearly 50% (Table 1).

**Table 1:** Sweet potato yield and disease incidence at different years.

Samples	Yield (t/hm^2^)	Disease incidence (%)
G1	30.76 ± 1.52	4.61 ± 1.23
G3	29.04 ± 1.13	23.2 ± 4.51
G5	26.78 ± 0.69	48.6 ± 7.22

### 3.5 Monoculture of sweet potato alters soil properties

Analysis of chemical parameters showed a slight decrease in soil pH and no change in total phosphorus (TP) content. In contrast, other parameters, including organic matter (OM), total carbon (TC), total nitrogen (TN), ammonium nitrogen (AN), nitrate nitrogen (NN), total potassium (TK), and available phosphorus (AP), all increased after continuous monoculture (Table 2).

**Table 2:** Chemical properties of bulk soil after consecutive monoculture of sweet potato.

Indices/samples	G1	G3	G5
pH	8.04 ± 0.02	7.66 ± 0.06	7.50 ± 0.01
OM (%)	0.89 ± 0.08	1.79 ± 0.04	2.05 ± 0.51
TC (%)	1.61 ± 0.04	2.69 ± 0.01	2.61 ± 0.03
TN (g/kg)	0.55 ± 0.01	1.35 ± 0.07	1.44 ± 0.04
AN (mg/kg)	24.86 ± 0.62	25.51 ± 0.52	31.04 ± 0.82
NN (mg/kg)	22.71 ± 0.33	23.07 ± 0.28	29.52 ± 0.29
TP (g/kg)	0.70 ± 0.03	0.73 ± 0.02	0.72 ± 0.22
AP (mg/kg)	18.32 ± 0.19	18.66 ± 0.13	21.99 ± 0.36
TK (g/kg)	10.05 ± 0.11	16.60 ± 0.12	16.26 ± 0.61

OM, organic matter; TC, total carbon; TN, total nitrogen; AN, ammonium nitrogen; NN, nitrate nitrogen; TP, total phosphorus; AP, available phosphorus; TK, total potassium.

### 3.6 Correlations between soil properties, cyanobacteria, and sweet potato yield

**Figure 4:**
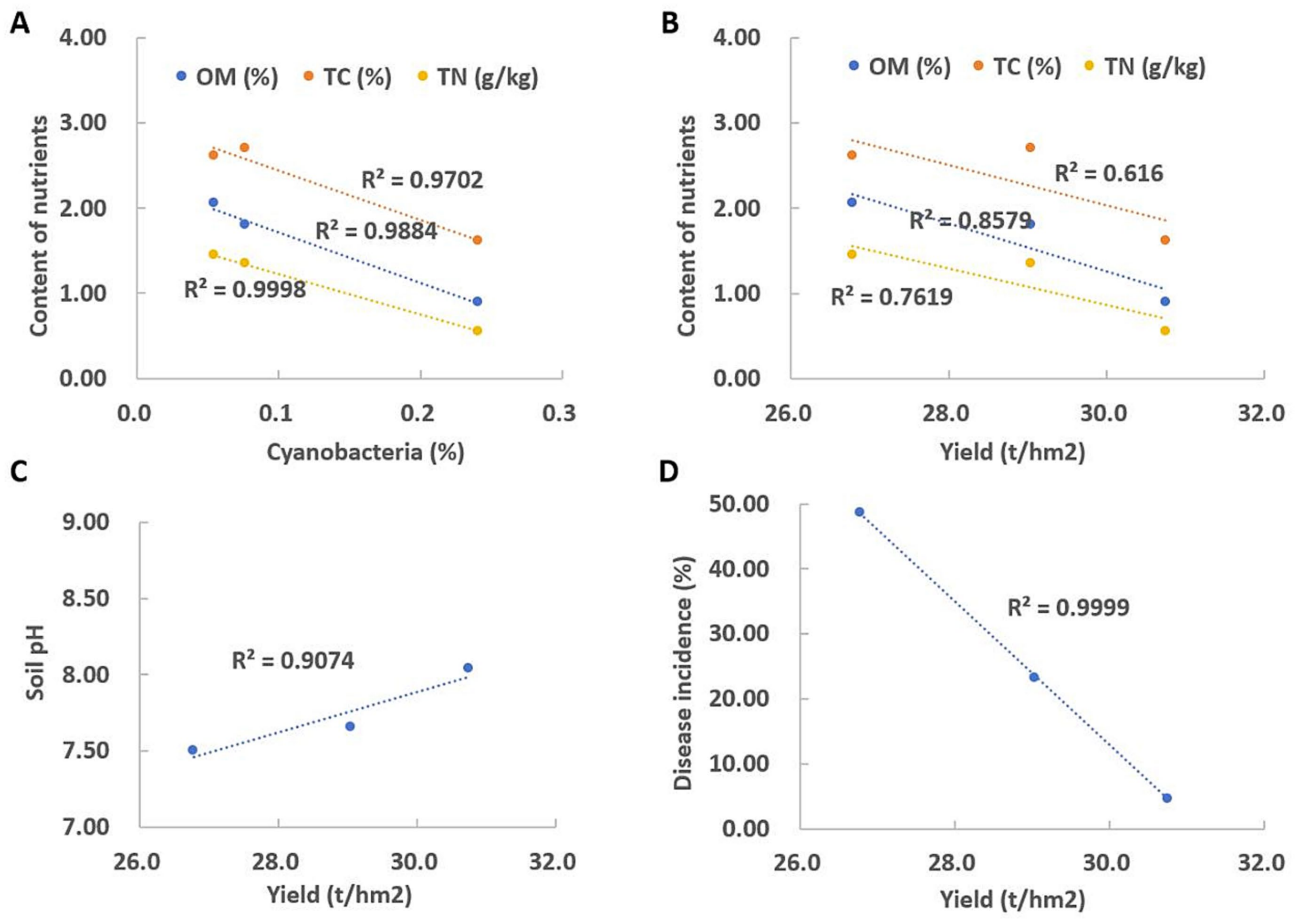
Correlation analysis between different indicators and yield. **(A)** Correlation between soil organic matter, total carbon, total nitrogen, and the abundance of Cyanobacteria. **(B)** Correlation between soil organic matter, total carbon, total nitrogen, and yield. **(C)** Correlation between soil pH and sweet potato yield. **(D)** Correlation between sweet potato yield and disease incidence.

As shown in Figure 4A, soil organic matter, total carbon, and total nitrogen showed good linear correlations with the abundance of Cyanobacteria (*R*^2^ > 0.9). However, these three nutrients showed weak negative correlations with yield (Figure 4B). Yield was positively correlated with soil pH, as lower yield was recorded under continuous monoculture when soil pH decreased (Figure 4C). A strong negative relationship was also observed between yield and disease incidence (Figure 4D).

## 4 Discussion

Due to its significant impact on sustainable agriculture, the soil microbiome is currently a hot topic that has attracted considerable interest (Babin et al., 2021). Characterization of soil microbiome communities and comparison of their structure are of great value for addressing challenges to agricultural sustainability (French et al., 2021). To harness the microbiome for precision management of sweet potato, especially for alleviating or even solving continuous cropping obstacles, the study was conducted using bulk soil samples collected from fields continuously cropped with the widely cultivated variety Shangshu 19 for 1, 3, and 5 years. Although three previous studies have conducted partially similar experiments, this study investigates the sweet potato-associated soil microbiome, including both bacteria and fungi at the same time, under longer-term consecutive monoculture. The sweet potato cultivar., sampling time points, and soil type all differ from those used in the three previous studies (Li et al., 2019; Gao et al., 2019; Gao et al., 2021).

A significant decrease in bacterial OTUs in soils after 3 and 4 years of continuous sweet potato cultivation has been reported (Li et al., 2019). In line with this previous report, our results showed a significant decrease in total richness of both bacteria and fungi, especially in the fifth year (Figures 2A,B). However, we observed fewer bacterial OTUs than reported in that study (e.g., 1,938 and 1,732 vs. 2,495 and 2,099 in the first and third years, respectively), which may be due to differences in temperature and soil type. Continuous cropping of sweet potato can lead to significant changes in the structure of bacterial and fungal communities in the soil (Li et al., 2019; Gao et al., 2019; Gao et al., 2021). Generally, it is characterized by a reduction in beneficial microorganisms and an increase in harmful ones, which, in turn, causes an imbalance in the soil microecology, intensifies the risk of soil-borne diseases, and reduces the yield and quality of sweet potato. This phenomenon is closely related to the continuous cropping of sweet potato and is one of the important causes of continuous cropping obstacles. The primary reason for the deterioration of soil microbial communities under continuous sweet potato cultivation is long-term monoculture, which disrupts soil ecological balance (Li et al., 2019). Specifically, this is manifested through the combined effects of multiple mechanisms, including the enrichment of pathogenic bacteria, suppression of beneficial microorganisms, accumulation of toxic root exudates, and deterioration of soil physicochemical properties. Microorganisms play a crucial role in organic matter decomposition, nutrient mineralization, and the nitrogen cycle. Bacterial- and fungal-feeding nematodes promote nitrogen mineralization by feeding on microorganisms, increasing the content of ammonium nitrogen and available nutrients in the soil. When microbial community diversity decreases, the transformation efficiency of soil nutrients such as nitrogen and phosphorus also declines, directly affecting crop growth. The decrease in specific microbial genera is largely caused by selective proliferation. This can happen due to selective inhibition by root exudates, pH-driven niche compression, carbon source deficiency, and pathogen competitive exclusion. Sweet potato continuously releases phenolic acids such as p-coumaric acid and ferulic acid, which directly inhibit the spore germination and mycelial growth of *Chaetomium* and *Trichoderma* but have no significant inhibitory effect on *Fusarium* (Choi et al., 2008). A decrease in soil pH can lead to a decline in neutral and slightly alkaline-preferring bacterial groups, such as *Gemmatimonas* and *Streptomyces*, due to the compression of their living space by the acidified environment. Saprophytic fungi such as *Chaetomium*, which rely on complex carbon sources, may be unable to maintain their populations due to insufficient energy. In contrast, pathogenic *Fusarium oxysporum* and *Verticillium dahliae* secrete iron carriers and extracellular polysaccharides to compete for space and nutrients, thereby forming a “pathogen-dominated” microecology.

Compared to the alteration trends observed in the rhizospheric soil of sweet potato, consecutive monoculture appears to have completely different impacts on the bulk soil microbiome. For example, the number of bacterial OTUs remained relatively constant during the first 3 years and even increased significantly in the fourth year (Gao et al., 2021). In another study, the same group reported variable changes in the number of fungal OTUs (from 314 to 642), with a significant decrease in the second year followed by an increase in the subsequent two years (Gao et al., 2019). Overall, the richness of both bacteria and fungi associated with sweet potato appears to be lower in bulk soil than in rhizospheric soil. In particular, only approximately half as many bacteria and one-third as many fungi were present in the third year in bulk soil compared to the rhizosphere. In fact, significantly fewer OTUs associated with different sweet potato cultivars grown in different types of soil were found in bulk soil than in the rhizosphere (Pepe-Ranney et al., 2020). This is quite reasonable because root exudates that attract soil microorganisms by inducing chemotaxis are mainly accumulated in the rhizosphere (Feng et al., 2018; Feng et al., 2021). In addition, soil microbiomes shared a much higher proportion of OTUs than the rhizospheric microbiome in the third year (1,458/1,732 and 96/177 vs. 507/3,207 and 86/513 for bacteria and fungi, respectively), suggesting a relatively stronger buffering effect and lower microbial heterogeneity in bulk soil after continuous cropping (Figures 2C,D).

Regarding the composition of the soil microbial community, we identified bacterial phyla similar to those reported previously, including Proteobacteria, Acidobacteria, Actinobacteria, Gemmatimonadetes, Firmicutes, and others (Li et al., 2019). As one of the most commonly found bacterial phyla across different plant-associated niches, Firmicutes—mainly composed of Gram-positive bacteria—are divided into three classes: *Clostridia*, *Bacilli*, and *Mollicutes* (Hashmi et al., 2020). A distinct decrease in Firmicutes under continuous monoculture of sweet potato was consistently observed, which may account for the lower abundance of Gram-positive phenotypes (Figure 3B). However, the lower abundance of facultatively anaerobic taxa seems to be due to a significant decrease in *Anaerolineaceae* within Chloroflexi, rather than in *Clostridium*, which is one of the largest genera within Firmicutes and is also typically found in anaerobic habitats (Figure 3D). For fungi, the most abundant phyla, including Ascomycota, Chytridiomycota, and Basidiomycota, were identified in both bulk and rhizospheric soils (Gao et al., 2019). At the genus level, the abundance of *Cryptococcus* significantly increased during continuous cropping of sweet potato in this study. A similar pattern has been reported in lily after consecutive replanting for 6 and 9 years (Shi et al., 2020). As an oligotrophic and pathogenic genus, *Cryptococcus* is commonly abundant in soil and is related to low-nutrient and oxygen-limited conditions; thus, it could be a potential indicator for monitoring and managing soil health sustainability. Research has shown that after continuous cultivation of sweet potato, the abundance of pathogenic fungi such as *Verticillium*, *Fusarium*, and *Colletotrichum* significantly increases, leading to frequent root and stem diseases and forming a vicious cycle in which “the more they are grown, the more diseased they become” (Gao et al., 2019; Gao et al., 2021). The deterioration of the soil microenvironment and the imbalance of the microbial community structure weaken the soil’s self-regulation ability, reduce its resistance and buffering capacity, and further inhibit the development of sweet potato roots and nutrient absorption. In addition, long-term plowing leads to a shallower plow layer (only 15–20 cm), forming a dense plow pan that hinders water and gas exchange, thereby causing oxygen deficiency in the root zone. At the same time, insufficient application of organic fertilizers reduces soil organic matter content, depriving microorganisms of carbon sources and energy and subsequently decreasing community diversity.

In contrast to previous studies that did not report potential pathogens, functional analysis using FAPROTAX revealed new findings in this study. First, soil bacteria showed an increase in human pathogens and animal parasites, largely due to increased abundance of *Nocardia*, *Roseomonas*, *Limnobacter*, *Caenimonas*, *Methylibium*, *Acinetobacter*, and *Stenotrophomonas* (Figures 3A,B). Although many species within these genera are well-known pathogens causing human/animal infections, none are considered to be related to plant diseases. Conversely, some of them, such as *Nocardia*, *Methylibium*, *Acinetobacter*, and *Stenotrophomonas*, have been reported to provide various benefits to plants (Nouioui et al., 2022; Santiago et al., 2017; Ishizawa et al., 2020; Ulrich et al., 2021). Nevertheless, these findings are worth noting, as they may have implications for farmers during field management. Second, we observed a significant decrease in *Cyanobacteria* with increasing duration of monoculture (Figure 3C). *Cyanobacteria* are ubiquitous photosynthetic prokaryotes that can improve soil nutrient status and water-holding capacity (Garlapati et al., 2019). They are oxygenic bacteria that play important roles in the global carbon and nitrogen cycles, especially by contributing to the nitrogen budget in diverse ecosystems (Liberton et al., 2019). Therefore, as proposed by others, inoculation with *Cyanobacteria* is considered valuable for the maintenance of soil fertility and health, and its application may also be effective in alleviating continuous cropping obstacles in sweet potato (Abinandan et al., 2019). Third, functions related to carbon metabolism, such as the degradation of hydrocarbons and aromatic compounds, were decreased (Figures 3D,E). On the one hand, a decrease in their degradation activity may lead to the accumulation of toxic pollutants in soil and exert adverse effects on soil health (Chakravarty et al., 2022). On the other hand, this decrease might promote an increase in Gram-positive aerobic bacteria, which are major contributors to hydrocarbon degradation (Lin et al., 2014). Consistent with the results obtained from community and phenotype analyses, the decrease in facultatively anaerobic bacteria such as *Pseudomonas* spp. and Gram-positive bacteria such as *Bacillus* spp. may account for the decreased degradation of hydrocarbons and aromatic compounds (Liu et al., 2020). In fact, a decrease in their abundance under continuous cropping has also been reported in potato (Zhao et al., 2020). Finally, functions related to nitrogen cycling were significantly changed (Figures 3F–I). For example, as an important index of soil fertility, the abundance of *nifH* (a nitrogenase-encoding gene) is positively affected by the diversity of plant species (Hao et al., 2022). In line with general expectations, we observed a decrease in microbial nitrogen fixation with increasing duration of monoculture. In addition, long-term N fertilization may also reduce microbial nitrogen fixation while stimulating nitrification. As reported in corn, we found that consecutive monoculture of sweet potato accelerates nitrification activity, potentially by increasing the abundance of ammonia-oxidizing bacteria (Huang et al., 2019). In addition, increased microbial nitrification leads to higher nitrous oxide emissions (a potent greenhouse gas) and potential contamination of ground and surface waters with nitrate (Wendeborn, 2020). In the continuous cropping system of sweet potatoes, there is a direct, quantitative, and clearly causal relationship between soil microbial functions, ecological processes, and agronomic traits. The core mechanism can be summarized as a three-level transmission pathway: “function driving—ecological response—phenotypic expression.” For example, *Streptomyces* and *Lysobacter* produce antibiotics such as actinomycin and chitinase, which directly inhibit *Fusarium oxysporum* and *Pectobacterium*. The decrease in their abundance is negatively correlated with the incidence of sweet potato stem rot disease (*R* = −0.81). Furthermore, the disease index increases by 0.83 units, resulting in a 12–18% decrease in the proportion of marketable potatoes for every 1% increase in *Fusarium* abundance.

There is a significant and quantifiable correlation between sweet potato yield and soil chemical parameters. Among these, soil pH, OM, NPK nutrient balance, and salt level are the key chemical factors affecting yield. As shown in Figure 4C, there is a significant positive correlation between pH and yield. A lower pH value inhibits the absorption of phosphorus, potassium, calcium, and magnesium, resulting in stunted plants. Continuous cropping often causes the soil to become acidic, significantly reducing the rate of root enlargement.

In conclusion, the study revealed a distinct succession in microbial community structure and demonstrated the significant impact of consecutive sweet potato monoculture on the health status of soil and nutrient cycling. In particular, functional analysis provided novel insights into the management or mitigation of continuous cropping obstacles in sweet potato. These data highlight the potential use of microbes such as *Cyanobacteria* and *Bacilli* as microbial inoculants for balancing C/N ratios and promoting sustainable sweet potato production. It is also worth noting the safety issues associated with continuous monoculture, including increased abundance of human pathogens and toxic pollutants in soil.

## Data Availability

The datasets presented in this study can be found in online repositories. The names of the repository/repositories and accession number(s) can be found in the article/Supplementary material.

## Footnotes

1
https://github.com/OpenGene/fastp

2
http://drive5.com/uparse/

3
https://www.arb-silva.de/

4
https://www.majorbio.com

5
https://bugbase.cs.umn.edu/index.html
