# Supplementary material for: Consecutive monoculture of sweet potato reduces yield due to deteriorated soil health and disrupted nutrient cycling
Source: Front Microbiol. 2026 Apr 30;17:1747390. doi: 10.3389/fmicb.2026.1747390 (PMC13171486; doi:10.3389/fmicb.2026.1747390)
Supplement: Supplementary file 1 [file Data_Sheet_1.docx]

# Supplementary Information

## Tables

Table S1 Summary of amplicon sequencing clean data of different samples

|  | **Bacteria** | | | **Fungi** | | |
| --- | --- | --- | --- | --- | --- | --- |
| **Sample** | **Seq_num** | **Base_num** | **Mean_length** | **Seq_num** | **Base_num** | **Mean_length** |
| S1_1 | 37607 | 16572211 | 441 | 33187 | 13294455 | 401 |
| S1_2 | 37450 | 16510174 | 441 | 34566 | 13844836 | 401 |
| S1_3 | 36889 | 16265946 | 441 | 44276 | 17742525 | 401 |
| S3_1 | 36870 | 16242894 | 441 | 31820 | 12748190 | 401 |
| S3_2 | 34314 | 15138797 | 441 | 37437 | 15000379 | 401 |
| S3_3 | 30938 | 13655293 | 441 | 31012 | 12415651 | 400 |
| S5_1 | 39797 | 17574788 | 442 | 30137 | 12080319 | 401 |
| S5_2 | 34233 | 15093738 | 441 | 41846 | 16770759 | 401 |
| S5_3 | 35194 | 15553880 | 442 | 44179 | 17701183 | 401 |

Table S2 List of 27 bacterial genera eliminated in both G3 and G5 after consecutive monoculture according to descending proportions presented in G1

| **Genus/Proportion (%)** | **G1-mean** | **G3-mean** | **G5-mean** |
| --- | --- | --- | --- |
| norank_f__MND8 | 6.446 | 0 | 0 |
| PAUC26f | 6.317 | 0 | 0 |
| Halomonas | 6.182 | 0 | 0 |
| BD1-7_clade | 5.471 | 0 | 0 |
| norank_c__Candidatus_Peribacteria | 2.661 | 0 | 0 |
| norank_c__Candidatus_Magasanikbacteria | 2.537 | 0 | 0 |
| Methylobacter | 2.454 | 0 | 0 |
| Algoriphagus | 2.437 | 0 | 0 |
| unclassified_f__Rhodobiaceae | 2.433 | 0 | 0 |
| Psychrobacter | 1.716 | 0 | 0 |
| Thauera | 1.693 | 0 | 0 |
| Chryseobacterium | 1.692 | 0 | 0 |
| norank_c__Candidatus_Woesebacteria | 1.58 | 0 | 0 |
| Methylobacillus | 1.575 | 0 | 0 |
| unclassified_f__Verrucomicrobiaceae | 1.565 | 0 | 0 |
| norank_p__RBG-1 | 1.461 | 0 | 0 |
| norank_p__Hydrogenedentes | 1.451 | 0 | 0 |
| norank_SubsectionIII | 1.328 | 0 | 0 |
| unclassified_c__Alphaproteobacteria | 1.217 | 0 | 0 |
| norank_f__cvE6 | 1.199 | 0 | 0 |
| norank_c__Candidatus_Pacebacteria | 0.9736 | 0 | 0 |
| norank_c__SPOTSOCT00m83 | 0.8519 | 0 | 0 |
| norank_f__SJA-28 | 0.7301 | 0 | 0 |
| norank_c__Gammaproteobacteria | 0.7286 | 0 | 0 |
| Vulgatibacter | 0.6072 | 0 | 0 |
| norank_f__Simkaniaceae | 0.6044 | 0 | 0 |
| unclassified_c__OPB35_soil_group | 0.6044 | 0 | 0 |

Table S3 Summary of functions among different groups predicted by the FAPROTAX

| **Function assignment/Proportion (%)** | **G1-mean** | **G3-mean** | **G5-mean** | **Record** |
| --- | --- | --- | --- | --- |
| chemoheterotrophy | 27.98 | 26.69 | 26.26 | 241 |
| aerobic_chemoheterotrophy | 25.08 | 23.35 | 23.71 | 210 |
| ***nitrification*** | ***8.165*** | ***10.26*** | ***11.34*** | ***54*** |
| aerobic_nitrite_oxidation | 4.102 | 4.961 | 6.79 | 34 |
| aerobic_ammonia_oxidation | 4.063 | 5.295 | 4.549 | 20 |
| ***nitrate_reduction*** | ***3.308*** | ***1.827*** | ***2.143*** | ***29*** |
| ***aromatic_compound_degradation*** | ***2.181*** | ***0.8447*** | ***0.4934*** | ***14*** |
| predatory_or_exoparasitic | 2.103 | 1.999 | 1.44 | 33 |
| sulfur_respiration | 2.079 | 2.545 | 2.826 | 39 |
| respiration_of_sulfur_compounds | 2.079 | 2.545 | 2.826 | 39 |
| chitinolysis | 1.93 | 2.44 | 1.76 | 8 |
| ***animal_parasites_or_symbionts*** | ***1.806*** | ***2.897*** | ***2.64*** | ***17*** |
| ***human_pathogens_all*** | ***1.75*** | ***2.873*** | ***2.63*** | ***15*** |
| ureolysis | 1.649 | 1.601 | 1.469 | 16 |
| ***human_pathogens_pneumonia*** | ***1.483*** | ***2.765*** | ***2.534*** | ***8*** |
| ***hydrocarbon_degradation*** | ***1.154*** | ***0.05806*** | ***0.1129*** | ***4*** |
| ***aromatic_hydrocarbon_degradation*** | ***1.027*** | ***0.05806*** | ***0.1129*** | ***2*** |
| fermentation | 0.8567 | 1.157 | 0.8753 | 14 |
| nitrate_respiration | 0.6767 | 0.5292 | 0.4668 | 10 |
| ***nitrogen_respiration*** | ***0.6767*** | ***0.5292*** | ***0.4668*** | ***10*** |
| ***nitrogen_fixation*** | ***0.6151*** | ***0.5419*** | ***0.4334*** | ***5*** |
| ***intracellular_parasites*** | ***0.4842*** | ***0.09456*** | ***0.1187*** | ***23*** |
| ***phototrophy*** | ***0.4819*** | ***0.3926*** | ***0.348*** | ***20*** |
| ***photoautotrophy*** | ***0.4168*** | ***0.3094*** | ***0.2447*** | ***15*** |
| manganese_oxidation | 0.3467 | 0.2005 | 0.3327 | 5 |
| cellulolysis | 0.3263 | 0.4791 | 0.3359 | 8 |
| nitrite_respiration | 0.2743 | 0.2821 | 0.2363 | 5 |
| nitrite_denitrification | 0.2481 | 0.2821 | 0.2363 | 4 |
| nitrous_oxide_denitrification | 0.2481 | 0.2821 | 0.2363 | 4 |
| nitrate_denitrification | 0.2481 | 0.2821 | 0.2363 | 4 |
| denitrification | 0.2481 | 0.2821 | 0.2363 | 4 |
| ***cyanobacteria*** | ***0.241*** | ***0.07601*** | ***0.05417*** | ***13*** |
| ***oxygenic_photoautotrophy*** | ***0.241*** | ***0.07601*** | ***0.05417*** | ***13*** |
| photoheterotrophy | 0.241 | 0.3166 | 0.2938 | 7 |
| ***methylotrophy*** | ***0.2123*** | ***0.05806*** | ***0.1181*** | ***9*** |
| methanol_oxidation | 0.1795 | 0.05806 | 0.1181 | 8 |
| anoxygenic_photoautotrophy_S_oxidizing | 0.1758 | 0.2334 | 0.1905 | 2 |
| anoxygenic_photoautotrophy | 0.1758 | 0.2334 | 0.1905 | 2 |
| xylanolysis | 0.1479 | 0.07901 | 0.05405 | 1 |
| dark_oxidation_of_sulfur_compounds | 0.05898 | 0.04892 | 0.02999 | 1 |
| nitrate_ammonification | 0.05665 | 0.04655 | 0.04385 | 1 |
| nitrite_ammonification | 0.05665 | 0.04655 | 0.04385 | 1 |
| aliphatic_non_methane_hydrocarbon_degradation | 0.05274 | 0.04445 | 0.1049 | 1 |
| methanotrophy | 0.03273 | 0 | 0 | 1 |
| dark_hydrogen_oxidation | 0.02482 | 0.01571 | 0.2633 | 2 |
| mammal_gut | 0.01078 | 0.007934 | 0.001951 | 1 |
| human_gut | 0.01078 | 0.007934 | 0.001951 | 1 |

## Figures


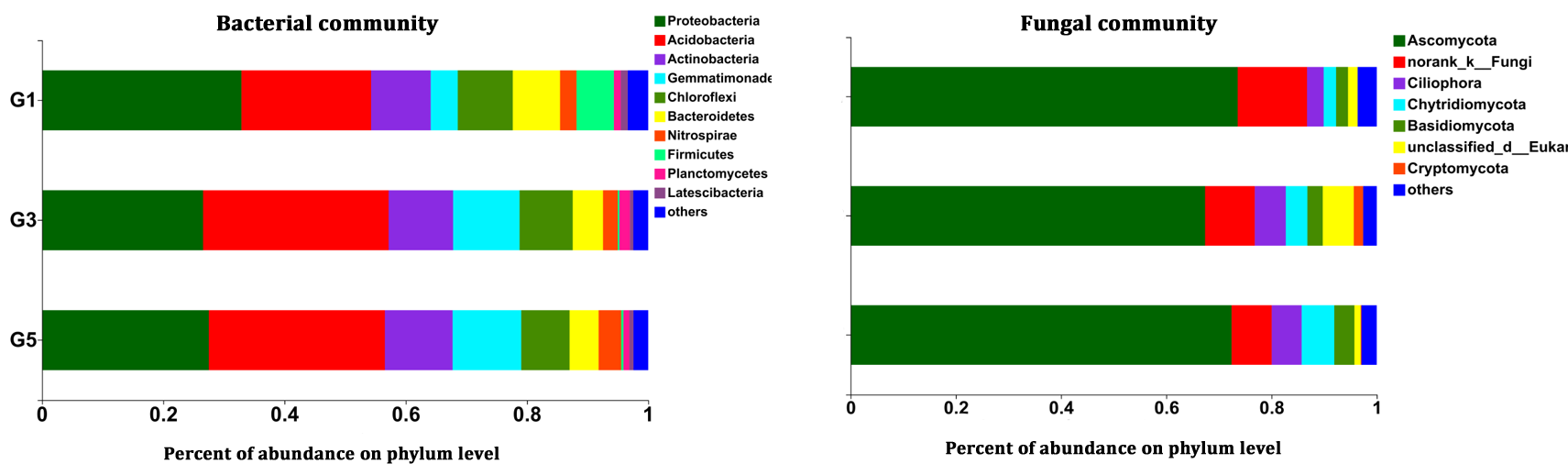


Fig. S1 Community barplot analysis of soil microbiome from three groups on phylum level


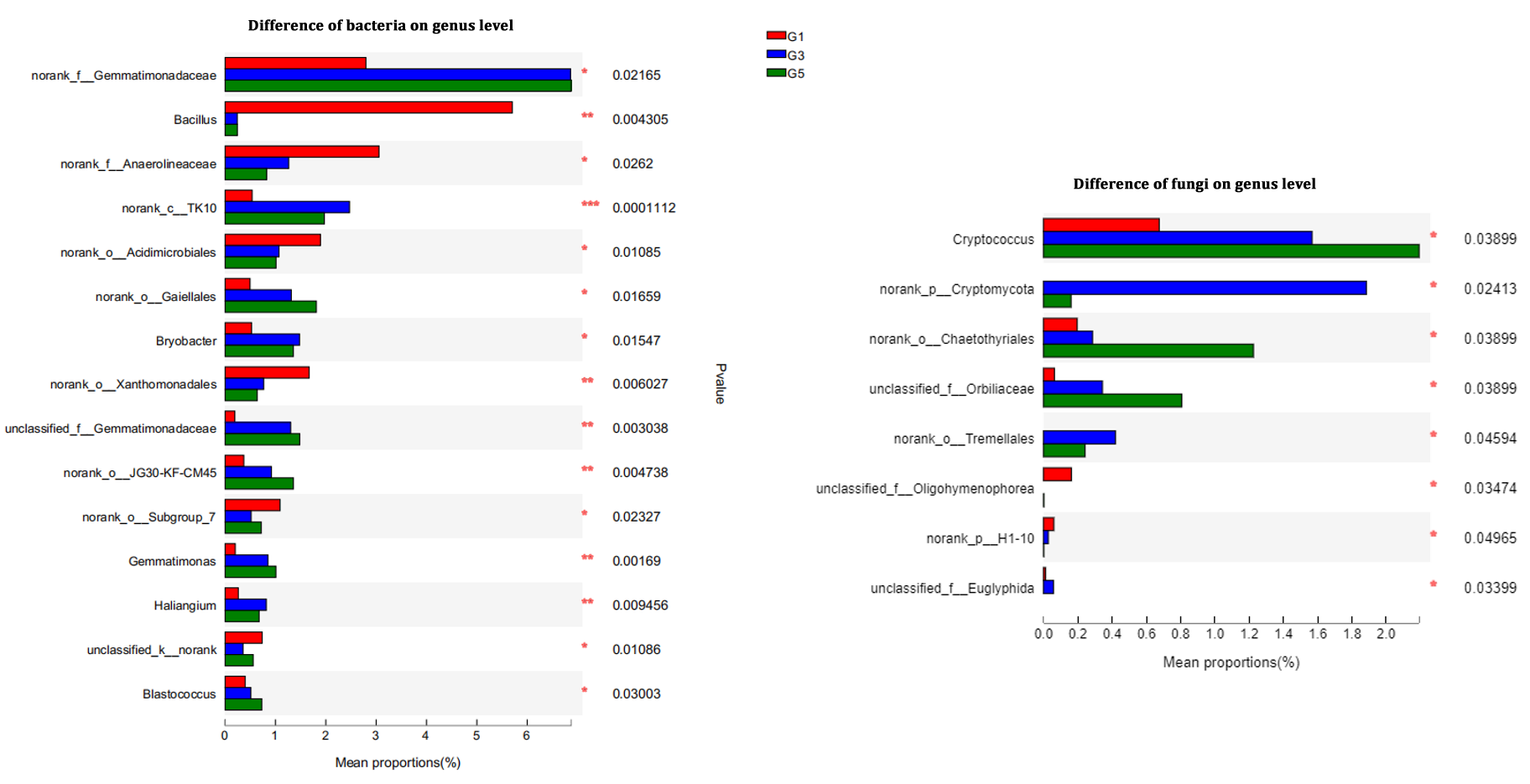


Fig. S2 Analysis of soil microbiome difference between three groups on genus level by Kruskal-Wallis test. *, 0.01 < P ≤ 0.05; **，0.001 < P ≤ 0.01; ***, P ≤ 0.001


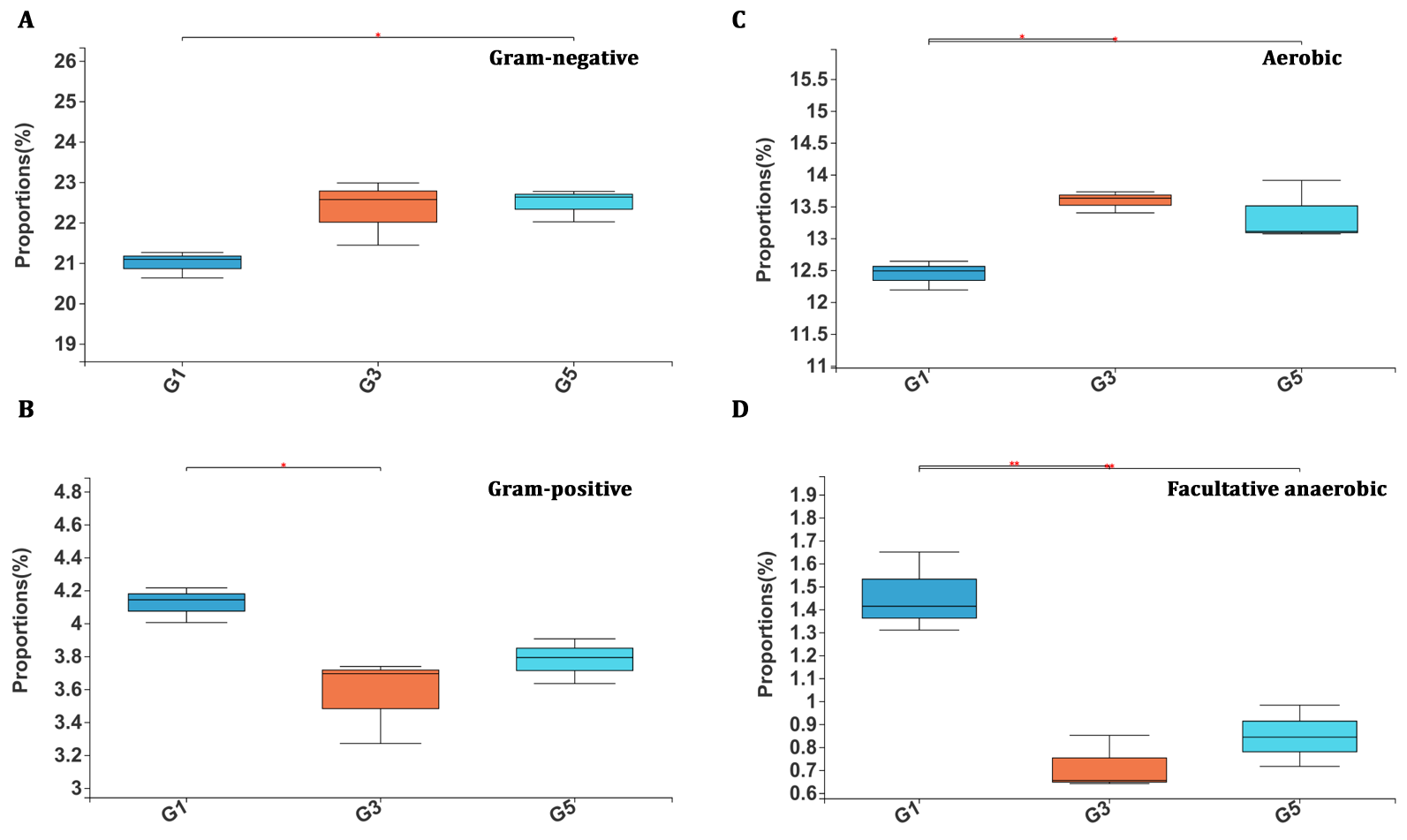


Fig. S3 Analysis of bacterial phenotype by BugBase among three groups on the genus level using Kruskal-Wallis H test. *, 0.01 < P ≤ 0.05; **，0.001 < P ≤ 0.01. (A) Relative phenotype abundance of Gram-negative. (B) Relative phenotype abundance of Gram-positive. (C) Relative phenotype abundance of aerobic. (D) Relative phenotype abundance of facultatively anaerobic.
